# Delayed presentation of congenital diaphragmatic hernia: a case report

**DOI:** 10.11604/pamj.2021.40.242.32113

**Published:** 2021-12-21

**Authors:** Saif Ghabisha, Faisal Ahmed, Saleh Al-wageeh, Ebrahim Al-shami, Abdu Al-hajri, Waleed Aljbri, Fawaz Mohammed

**Affiliations:** 1 Department of General Surgery, Ibb University of Medical Science, Ibb, Yemen,; 2 Urology Research Center, Al-Thora General Hospital, Department of Urology, Ibb University of Medical Science, Ibb, Yemen,; 3 Department of Urology, School of Medicine, 21 September University, Sana'a, Yemen,; 4 Department of Orthopedy, Ibb University of Medical Science, Ibb, Yemen

**Keywords:** Congenital diaphragmatic hernia, late presentation, surgery, case report

## Abstract

Congenital diaphragmatic hernia (CDH) is known as a structural defect caused by inadequate fusion of the pleuroperitoneal membrane forming the diaphragm, allowing peritoneal viscera to protrude into the pleural cavity. It affects nearly one out of 2500 live births. We here report the case of a six-month-old boy with left diaphragmatic hernia presenting with poor feeding, breathing difficulty, cough, and recurrent pneumonia in the last 2 months. Chest X-ray and computed tomography scan revealed left sided CDH. The defect was corrected through open surgical repair without complications. At 5-month follow-up a radiograph was performed which revealed full recovery. The primary goal of this report was to alert physicians to suspect this diagnosis in patients with unexpected presentation of diaphragmatic hernia.

## Introduction

Congenital diaphragmatic hernia (CDH) is a hereditary congenital disorder characterized by abnormal diaphragmatic growth, which affects one out of 2500 neonates and has an overall survival of 67%. This condition is caused by a diaphragmatic tunnel that lets peritoneal viscera protrude into the pleural cavity [1]. Congenital diaphragmatic hernia is most commonly associated with neonatal respiratory distress; however, late-presenting CDH has minimal side effects and a better prognosis [2]. Delay in CDH presentation has been observed in 2.5-20% of all CDH patients. Nonetheless, the factors associated with late CDH presentation are mischaracterized [3]. Patients with prolonged respiratory and gastrointestinal symptoms of unknown etiology might be suffering from this disease. Due to its milder and more perplexing clinical presentation, this type of CDH poses a significant diagnostic challenge [4]. Herein, we presented a case of a diaphragmatic hernia in a six-month-old boy. The manifestations, diagnosis, and outcome are discussed.

## Patient and observation

**Patient information:** a 6-month-old boy with chief compliant of low-grade fever, poor feeding, intermittent respiratory distress, cough, and gastrointestinal symptoms such as nausea, vomiting for about 2 months ago; his condition was initially noticed when he was two weeks old. His mother gave a history of repeated aspiration while feeding and repeated hospital admissions three times in the past for suspected aspiration pneumonia. He also had a history of severe cough while feeding, on several occasions.

**Clinical findings:** regarding the physical examination, the pulse rate was 72 beats per minute, the respiratory rate: 26 per min, the O_2_ saturation: 98%, and the oral temperature: 37.8°C. The birth weight was 3200 g, and the current weight was 6.5 kg. The patient was also moderately dehydrated; there was less air entrance in the left lung, and the heart sounds mainly were noticed on the right lung. The abdomen was scaphoid without any palpated mass or tenderness. Additionally, the bowel sound was noticed in the left thoracic cavity.

**Figure 1:**
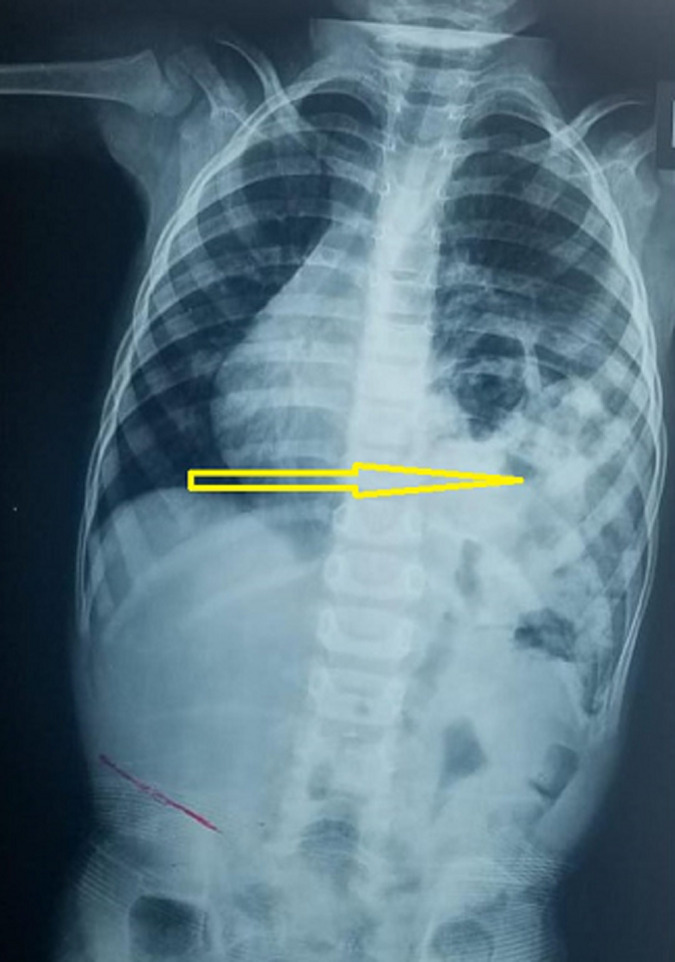
plain chest X-ray shows the left diaphragmatic hernia (arrow)

**Figure 2:**
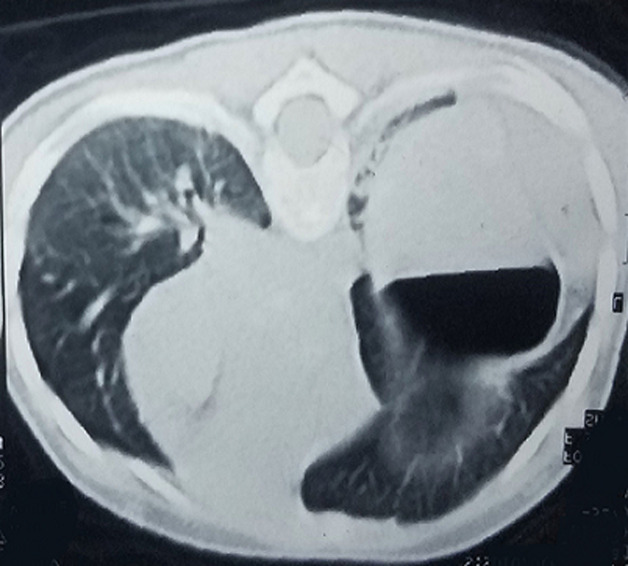
a chest computed tomography scan reveals a large diaphragmatic defect in the left side and complete collapse of the left lung

**Diagnostic assessment:** according to the chest X-ray, the stomach was seen in the left chest cavity with right-shifted mediastinum and confirmed the diagnosis of left CDH (Figure 1). Chest and abdominal computed tomography (CT) scans were carried out for validating the diagnosis of CDH and recommended collapse of the left lung and the right-shifted mediastinum (Figure 2). Following the baseline assessment, the patient was transferred to a pediatric surgical center.

**Therapeutic interventions:** via general anesthesia with supine position, the abdomen was opened through the left subcostal incision, where a left diaphragmatic defect of around 5 cm in diameter was detected. The existence of the small bowel, descending colon, stomach, and spleen within the chest cavity was observed and necessitated a decrement of the components to the peritoneal cavity. In addition, the hernial defect was repaired with “U” shape threads. Following confirmation of diaphragmatic consistency, the chest tube was placed, and the operation was ended without intraoperative complications.

**Follow-up and outcome:** a control chest X-ray was performed 24 hours after the operation, which revealed a cardiac silhouette in a proper position and good expansion of the left lung, as well as right lung infiltration. The chest tube was removed on the 9^th^postoperative day. The child was discharged from the hospital on the 11^th^ postoperative day without complications. After a 5-month follow-up, the patient was fine, and the chest X-ray was normal. The study of abnormalities in the genetic pattern was halted due to budget reasons and lack of such investigations in our city.

## Discussion

Hunter and Mc Cauley published the first explanation of the pathophysiology of CHD in 1754, defining it as the protrusion of peritoneal organs into the pleural cavity or the descendants of thoracic organs into the peritoneal cavity as a consequence of the lack of closure of the pleuroperitoneal canals. The defect is more prevalent on the left side (80% of the time) and less common on the right (the rest of the time); bilateral ones are pretty uncommon [5]. Abdominal organs may migrate into the thoracic cavity via the diaphragmatic defect, which leads to lung compression and alteration of the mediastinum position to the opposite direction. Depending on the extent of pulmonary compression, there may be a marked decline in pulmonary branches, restricted development of alveoli, and muscle hypertrophy in lung arterioles, leading to a reduced functional lung capacity or pulmonary hypoplasia [6].

The clinical features of CDH with late presentation comprise a wide range, in which gastrointestinal symptoms (such as vomiting and abdominal pain) and lung problems (such as shortness of breath, cough, and tachypnea) could appear alone or together [4]. Our patient was presented with gastrointestinal and respiratory symptoms. During a physical examination, an excavated abdomen (hallow-out area), displacement of heart sounds, and auscultation of bowel movements in the chest may be seen [3]. Cardiovascular and respiratory manifestations are typically caused by thoracic organ compression as well as other congenital chest anomalies [1]. When the diagnosis is ambiguous, the spiral chest CT scan, plain chest X-ray after placement of a nasogastric tube, and/or Barium swallow could be performed to rule out the occurrence of CDH [4]. Our patient's diagnosis was carried out according to a senior radiologist's review of his chest X-ray.

Late-presenting CDH is estimated to account for 2.5% to 20% of all CDH cases [3]. It might be hard to diagnose due to the rarity of early presentation in the outpatient clinics. It is frequently overlooked, which can lead to misdiagnosis of radiographic imaging. Previous reports have shown late-presenting CDH being misdiagnosed as effusion of pleura, lung infection (pneumonia), pneumothorax, pneumatocele, and lung abscess [7-9]. Having a history of normal antenatal ultrasounds and previous normal chest radiograph can be additional confounding factors to make an accurate diagnosis [9]. Bagaj found that 25% of late-presenting CDH in children was not diagnosed or was inaccurately diagnosed in a review of 122 articles and on 349 children [7]. While Elhalaby *et al*. reported that in their work, three out of the 15 patients who presented after two months of birth were incorrectly managed with chest drainage [10]. Our patient had a previous history of repeated hospital admission with inaccurate diagnosis and he was treated as aspiration pneumonia. Regarding late-presenting CDH, morbidity and mortality still remain high. Research has shown a mortality rate of 40-60% [4]. Bronchopulmonary sequestration, lung cystic adenomatoid deformity, and bronchogenic cysts are all possible differential diagnoses of CDH [10].

Principal or patch closure of the diaphragm via an open abdominal technique is usually used for surgical repair. Whenever the diagnosis is overdue on account of doubts of visceral-chest adhesions, a thoracotomy or combination of thoracic and abdominal method is considered [11], as reported in our case. Previous studies have reported the thoracoscopic method to be successful, but it is tainted with a high risk of recurrence. Moreover, during thoracoscopy procedure, the increase in pulmonary pressure with subsequent hemodynamic instability may develop; additionally, the placement of patch via thoracoscopy may increase the time of operation [12]. Given all these explanations, in the presence of small CDH and/or mild pulmonary hypertension, thoracoscopic repair of CDH could be recommended. Lately, the laparoscopic technique for CDH has been reported to be effective and safe and may be an excellent choice [12]. Our case had a good outcome, and the favorable factors of our patient were as follows: the CDH was isolated, the diaphragmatic defect was moderate (approximately 5 cm), the hernia was on the left side, and the liver was still in the abdomen [1].

Even though our patient did not have co-existing congenital defects according to CT and ultrasonography findings, it is essential to look out for other anomalies in any CDH patient. Heart defects at birth were the utmost common defect, followed by urinary tract anomalies, spinal cord deformity, Pierre-Robin syndrome, atresia of the esophagus, omphalocele, and choledochal cyst [9]. According to Elhalaby *et al*. 40% of children with late presentation of CDH had malrotation of the midgut, 7% had polycystic kidney disease, and 7% had a deformity in spinal cord [10]. However, the study of abnormalities in the genetic pattern in our patient was halted due to budget reasons and lack of such investigations in our city.

**Patient perspective:** the patient's family was happy with the successful outcome of the surgery.

**Informed consent:** a written informed consent was obtained from the patient's family for participation in our study.

## Conclusion

The absence of usual clinical presentations in cases of late-presenting CDH affects the diagnosis of this defect. In every child with chronic unresponsive respiratory difficulties and GI disturbances, late CDH should be considered through different methods of diagnosis, and radiologic imaging studies are required. Additionally, the open surgical repair via the abdominal approach was the best option for our patient.
